# Anxiolytic and Antidepressant-Like Effects of *Conyza canadensis* Aqueous Extract in the Scopolamine Rat Model

**DOI:** 10.3390/plants10040645

**Published:** 2021-03-29

**Authors:** Jamila El-Akhal, Ioana Humulescu, Radu Ionita, Paula Alexandra Postu, Eugen Ungureanu, Monica Hancianu, Rachid Bencheikh, Silvia Robu, Oana Cioanca, Lucian Hritcu

**Affiliations:** 1 Bioactive Molecules Laboratory, Faculty of Sciences and Technologies Fez, Sidi Mohamed Ben Abdellah University, B.P. 2202 Fez, Morocco; jamila.elakhal@usmba.ac.ma (J.E.-A.); rachid.bencheikh@usmba.ac.ma (R.B.); 2 Faculty of Pharmacy, “Grigore T. Popa” University of Medicine and Pharmacy, 16 Universitatii Street, 700115 Iasi, Romania; ioana.humulescu@umfiasi.ro (I.H.); monica.hancianu@umfiasi.ro (M.H.); 3 Department of Biology, Alexandru Ioan Cuza University of Iasi, Bd. Carol I, No. 11, 700506 Iasi, Romania; radu.ionita@uaic.ro (R.I.); paulaalexandra.postu@gmail.com (P.A.P.); aeu@uaic.ro (E.U.); hritcu@uaic.ro (L.H.); 4 Faculty of Medicine and Pharmacy, “Dunarea de Jos” University, 35 Al. I. Cuza Street, 800010 Galati, Romania; silvia.robu@ugal.ro

**Keywords:** *Conyza canadensis*, catechins, flavonoids, antioxidants, anxiety, depression, scopolamine

## Abstract

*Conyza canadensis* is a plant widely used in traditional medicine in Morocco for the treatment of varied health challenges. However, to the best of our knowledge, there is no scientific study justifying the traditional use of *Conyza* extract as an anxiolytic and antidepressant agent. Moreover, data regarding the polyphenolic fraction is limited. Therefore, the present study was conducted to investigate the chemical composition of an aqueous extract obtained from the aerial parts of *Conyza*, its antioxidant potential, and the anxiolytic and antidepressant-like effects of the sample (100 and 200 mg/kg body weight (bw)) in the scopolamine (Sco) (0.7 mg/kg bw) rat model. To achieve this purpose, a variety of antioxidant tests (including free radical-scavenging activity and lipoxygenase-inhibitory potential assays) and behavioral procedures, such as the elevated plus-maze and forced swimming tests, were performed. The results demonstrated that the aqueous extract of *Conyza canadensis* is rich in catechins and flavonoids which possess good antioxidant activity. Additionally, concentrations of 100 and 200 mg/kg of the extract exhibited significant anxiolytic and antidepressant-like profiles following scopolamine treatment. Therefore, we propose that the use of *Conyza canadensis* could be a new pharmacological target for the amelioration of major depression.

## 1. Introduction

Plants have been one of the important sources of medicines since the beginning of human civilization [1]. Historically, natural products have been used in most cultures, since ancient times, for the treatment of many diseases and illnesses. Despite huge progress in the health system, the use of herbal remedies for the prevention and treatment of diseases remains popular. Recently, the search for plant-based drugs has attracted increased scientific attention. There exists considerable growing interest in the discovery of new natural sources that may offer a useful alternative, or complementary choice, in either the prevention or the treatment of various health challenges. It has been demonstrated that natural products are preferable to synthetic compounds. The numerous side effects of synthetic drugs have led to the use of medicinal plants as a reliable source of new therapies [2].

Although many natural products have been described to possess several therapeutic benefits, many others remain partially or fully untested and their use is not monitored [3]. It becomes a necessity, therefore, to perform further studies to ascertain the efficiency and safety of these herbal remedies—as well as to provide scientific proof in animal models for the traditional utilization of these herbal remedies in folk medicine. 

*Conyza canadensis* (CC) belongs to the well-known Asteraceae family [4]. This annual, biennial, or perennial plant seems to grow well in very damp ground. In ground submerged in freshwater it rarely shrubs, growing 1–2 m tall. The stems are erect and branched with alternating leaves. It is mostly considered a weed, but the traditional medicine of various countries of origin indicates its utility in treating gastrointestinal problems.

CC is reported to have massive biological activities, encompassing antiproliferative activity [5], effect on gastrointestinal problems—most commonly diarrhea and dysentery, and as a diuretic agent [6]. It is also reported to be useful in the treatment of internal hemorrhages, gonorrhea, and bleeding piles [7]. A literature survey revealed that the whole plant is antirheumatic, astringent, balsamic, emmenagogic, styptic, tonic, and vermifuge [8] and that its leaves are experimentally hypoglycemic [9]. CC was reported to exhibit a significant anti-inflammatory effect on rats with carrageenan- and formalin-induced edema [10]. However, most of the chemical studies were oriented on the essential oil isolated from various organs of this plant. Thus, the present study was designed to assess the chemical composition and the antioxidant activity, as well as the anxiolytic and antidepressant-like effects, of an aqueous extract of CC in the scopolamine (Sco) rat model.

## 2. Results and Discussion

### 2.1. Chemical Analysis of the Investigated Extract

**Figure 1:**
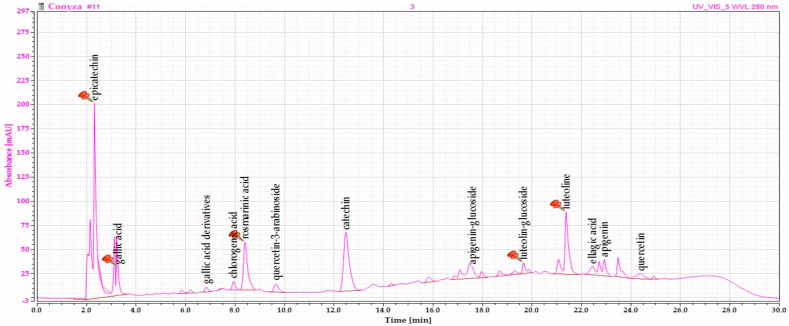
Chromatogram of the *Conyza canadensis* (CC) aqueous extract.

The chromatographic identification of the active compounds was assessed using a fast liquid chromatography method which lasted up to 30 min. The general aspect of the HPLC-RP chromatogram is shown in Figure 1, and it comprises the main compounds that were found and quantified in the investigated aqueous extract. Compared with all published data on *Conyza canadensis*, our research is original due to the type of plant material (the aerial parts) and the extract obtained using water as a solvent.

To date, the scientific literature is rich in data and research regarding the chemical composition of the essential oil extracted from *C. canadensis* roots and aerial parts, but other references to the polyphenolic derivatives existent in this species are scarce. The extract included in this study is an aqueous solution which justifies the primarily extraction of catechins, flavone glycosides and polyphenolic acids. The choice of this type of extraction was based on the general use of infusions or brewed teas in the general population. Moreover, data regarding such extracts obtained from CC lacks in international databases. At the time of writing, only four articles refer to the polyphenols from CC [11,12,13]. Some only quantify the total amount of flavonoids, polyphenols, and tannins and measure the antioxidant and antimicrobial activity of the extracts. However, Liu et al. [14] identified six compounds from CC referred to as eugenyl beta-Psd, scutellarin, luteolin-7-O-beta-D-glucuronide, quercetin, quercetin-3-O-beta-D-glucopyranoside, and luteolin. Our results are similar to some extent, but one should never forget that the pedoclimatic conditions have a significant impact on the biosynthetic capacity of plants. The plant material included in our study was harvested from a dry tropical climate, in contrast to the humid subtropical climate of the area from which other investigated materials were harvested. However, the found components are mainly flavonoids such as catechin and epicatechin, luteolin and its glucoside, apigenin, and apigenin-7-O-glucoside, as well as quercetin and quercetin-3-arabinoside. Some phenolic acids were also identified, but their quantity amounted to less than a third (45.020 µg/mg dry extract) of the flavonoid fraction (Table 1).

### 2.2. In vitro Antioxidant Potential Evaluation

The antioxidant potential of plant extracts is well recognized and can be evaluated by various means [15]. In our research, we used some of the most commonly employed tests, which enabled us to comprehend the true potential of the newly investigated *C. canadensis* compared with other extracts with similar chemical composition but obtained from other plant species. The obtained values indicated that CC extract can chelate iron and scavenge DPPH radical. Although DPPH radical is a synthetic compound that is available only for lab testing, its use in assessing antioxidant capacity is accepted worldwide. Moreover, the intensity of lipoxygenase (LOX) inhibition at concentrations above 5 mg/mL is comparable with quercetin, which was used as a standard. Considering that the extract is mainly constituted by flavones and catechins, these compounds have free hydroxyl and carbonyl moieties, which increase the capability to chelate metals, ions, and radicals. Flavonoid-rich extracts or fractions demonstrated excellent antioxidant activity in both in vitro and in vivo tests [15,16,17,18]. 

**Figure 2:**
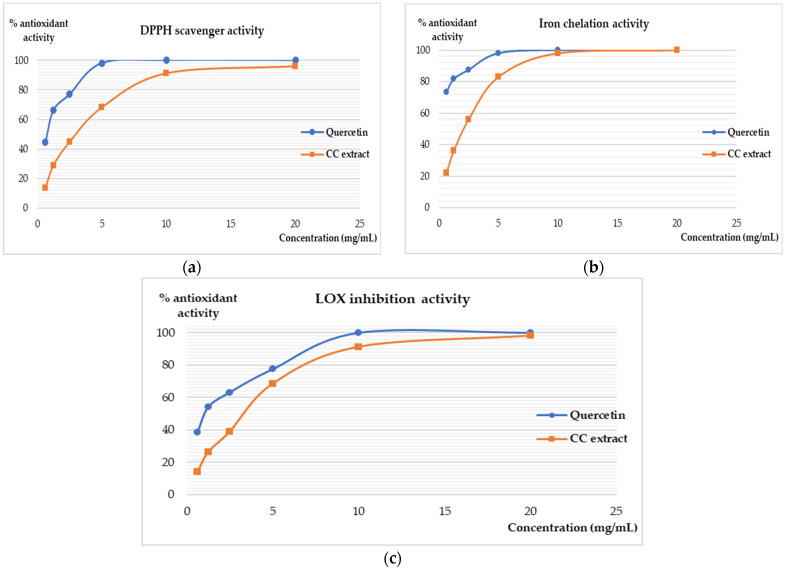
The in vitro antioxidant activity of CC extract. (**a**) DPPH radical scavenger assay; (**b**) iron chelation activity test; (**c**) lipoxygenase (LOX)-inhibitory potential.

As indicated in Figure 2, the CC extract decreased the quantity of free DPPH radicals in a direct concentration-dependent manner, but it never reached 100% activity, with a value of 126.03 µg/mL for IC_50_ (inhibitory concentration for 50% of the radicals present in the tested solution). The intensity of our sample is lower than the efficacy of quercetin. However, this is not uncommon for crude plant extracts when compared with a single pure compound. For the obtained values, the comparison with the standard is statistically significant (*p* = 0.0161). Thabit at al. [12] indicated that the ethanolic extract obtained from the whole plant (*Conyza bonariensis*) has good antioxidant and antimicrobial potential. Although the species are related, the obtained results are different from the literature; the solvent used in this case was ethanol, which has a better extractability for some lipophilic compounds (aglycons, volatiles, etc.) than water—our chosen solvent. 

Compared with DPPH, iron is normally found in both its forms in live organisms and is implicated in the Fenton reaction, which generates hydroxyl radicals. The most aggressive form is Fe^2+^, which is a source of oxidative stress and free radical overproduction. Therefore, natural compounds that can chelate such iron forms are of essential importance for their protective potential. Our results indicated that, at concentrations of 10 mg/mL, CC extract has the same intensity of action as the standard (Figure 2b). The IC_50_ value was closer to that of quercetin (42.97 µg/mL for CC extract vs. 18.47 µg/mL for quercetin). The activity is correlated with the concentration and statistically significant (*p* < 0.0001). This signifies that the investigated sample has a stronger affinity to iron chelation than DPPH radicals.

The antioxidant potential was also assessed in the lipoxygenase (LOX) assay. LOX represents a family of enzymes distributed in the living world. They are implicated in lipid oxidation and peroxidation processes, resulting in energy and free radicals [16,19]. A similar inhibition curb profile is noted for the CC extract and the standard (*p* < 0.0001). The IC_50_ value for quercetin was 17.46 µg/mL, whereas 49.22 µg/mL was calculated for the sample. Such values sustain the hypothesis that CC extract is efficient in inhibiting LOX activity. Considering that lipoxygenase needs a substrate for its activity, iron chelation prevents the existence of such substrates. Due to its reversible transformation from Fe^3+^ to Fe^2+^, iron chelation represents an indirect mechanism of control of LOX activity. Our study is the first to use iron chelation and LOX assays to evaluate the antioxidant potential of the polyphenols found in *Conyza canadensis*. The efficacy of our sample was compared with previous research on other plant species (*Matricaria chamomilla*, *Pelargonium* sp., *Ocimum* sp.) with a similar chemical composition [16,17,18]. Therefore, all assays’ results are consistent and prove the antioxidant activity of the investigated extract.

The type of flavonoid that is predominant in CC aqueous extract is a determinant for the biological effects [15]. Both catechins and flavone glycosides can reduce the reactive oxygen species primarily by two mechanisms which are related to the assays included in our research. The results are in agreement with other data, which indicate that flavonoid compounds can suppress the activity of various enzymes such as LOX, cyclooxygenase, monooxygenase, and glutathione S-transferase. Moreover, the same compounds can chelate metal ions, which are generators of free oxygen radicals. Overall, the aqueous CC extract has good antioxidant activity with multiple modes of action which indicate that this extract could be used in further investigations [15].

### 2.3. In vivo Anxiety Assessment in the Elevated Plus-Maze Test

**Figure 3:**
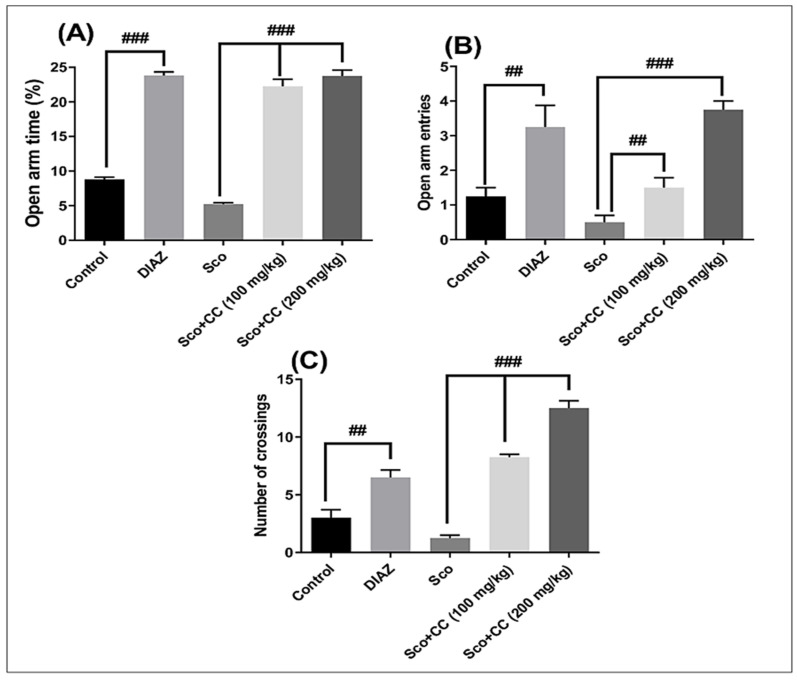
Effects of the *Conyza canadensis* (CC) aqueous extract (100 and 200 mg/kg body weight (bw)) in an elevated plus-maze test on: (**A**) the percentage of the time spent in the open arms, (**B**) the number of open-arm entries, and (**C**) the number of crossings in scopolamine (Sco, 0.7mg/kg bw)-treated rats. Values are means ± standard error of the mean (SEM) (n = 6 animals per group). For Tukey’s post hoc analyses: ## *p* < 0.001 and ### *p* < 0.0001.

As illustrated in Figure 3A, Sco exposure significantly decreased the time spent in the open arm (*p* < 0.001), as compared with the control group. Rats given Sco with CC extract at 100 and 200 mg/kg significantly increased the time spent in the open arm (*p* < 0.0001), as compared with the Sco-alone-treated group. The number of entries in the open arm (Figure 3B) was significantly (*p* < 0.001) reduced by Sco, whereas administration of the CC extract significantly increased the number of entries in the open arms, especially at the dose of 200 mg/kg, as compared with the Sco-alone-treated group. 

As seen in Figure 3C, Sco-injection-induced hypolocomotion, as evidenced by a decreased number of crossings (*p* < 0.001), as compared with the control group. Treatment with the CC extract significantly increased locomotion (*p* < 0.0001), as compared with the Sco-alone-treated group, suggesting an anxiolytic profile. According to a literature survey, several phytochemical studies reported that CC contains terpenes, acetylene derivatives, flavonoids, benzoic acid derivatives, alkaloids, essential oils, sphingolipids, fatty acids, and sterols [5,20]. Among them, it has been reported that quercetin protects against stress-induced anxiety- and depression-like behavior and improves memory in male mice [21]. Rai et al. [22] concluded that catechin ameliorated depressive symptoms in Sprague Dawley rats, subjected to chronic unpredictable mild stress, by decreasing oxidative stress. In addition, Stringer et al. [23] demonstrated that epicatechin mitigated anxiety in association with elevated hippocampal monoamine and BDNF (brain-derived neurotrophic factor) levels, but did not influence pattern separation in mice. Kumar et al. [24] reported that apigenin 7-glucoside from *Stachys tibetica* Vatke ehxibited anxiolytic effect in rats. Crupi et al. [25] demonstrated for the first time that the luteolin compound exerts a significant antidepressant effect at a low dose and may be considered as a novel therapeutic strategy in depression. Samad et al. [26] demonstrated the protective effect of gallic acid against arsenic-induced anxiety- and depression-like behaviors and memory impairment in male rats. Miyazaki et al. [27] demonstrated that chlorogenic acid showed behavioral pharmacological anxiolytic activity and activation of hippocampal BDNF-TrkB (brain-derived neurotrophic factor and tropomyosin receptor kinase B) signaling. Rosmarinic acid exhibited anxiolytic effects in a beta-amyloid 1-42 (Aβ1-42)-induced mouse model of Alzheimer’s disease as reported by Mirza et al. [28]. Girish et al. [29] demonstrated that administration of ellagic acid to mice produced anxiolytic effects when tested in the elevated plus-maze. The authors suggested the involvement of the GABAergic system (responsible with the synthesis and degradation of γ-aminobutyric acid) in the anxiolytic-like effect of ellagic acid in mice.

Our findings demonstrated that the CC extract eliminated the anxiogenic effects of Sco, acting as an anxiolytic agent with similar potential to diazepam (DIAZ), a typical anxiolytic agent.

The obtained results are in direct correlation with the chemical composition and the in vitro antioxidant activity. Moreover, CC extract administration does not have a sedative effect, nor the side effects usually seen in DIAZ. The increased number of crossings proves that the animals are not only less anxious but also more active than the positive control group. Translated to further studies, this may relate to an increased standard of living for mentally impaired persons. However, this needs to be investigated in future research.

### 2.4. In vivo Depressive Response Evaluation in the Forced Swimming Test

**Figure 4:**
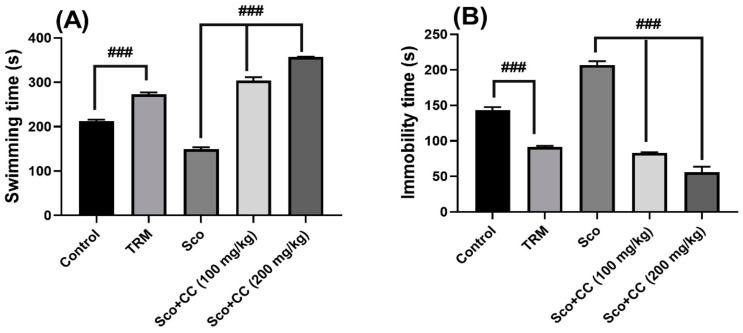
Effects of the *Conyza canadensis* (CC) aqueous extract (100 and 200 mg/kg bw) in the forced swimming test on: (**A**) the swimming time, and (**B**) the immobility time of scopolamine (Sco, 0.7mg/kg bw)-treated rats. Values are means ± SEM (n = 6 animals per group). For Tukey’s post hoc analyses: ### *p* < 0.0001.

The results from the forced swimming test (FST) are illustrated in Figure 4A,B. According to Figure 4, scopolamine exposure induced depression-like behavior as evidenced by decreased swimming time (*p* < 0.01) (Figure 4A) and increased immobility time (*p* < 0.01) (Figure 4B), as compared with the control group. Treatment with CC extract (100 and 200 mg/kg) in rats given Sco caused a significant increase in the swimming time (*p* < 0.0001) (Figure 4A) that paralleled a significant decrease in the immobility time (*p* < 0.0001) (Figure 4B), suggesting an antidepressant profile. 

The antidepressant profile of the CC extract is supported by the chemical constituents identified—(epi)catechin, luteolin, rosmarinic acid, gallic acid, and quercetin—in its chemical composition, as previously stated. Lee et al. [30] reported that chronic administration of catechin decreased depression- and anxiety-like behaviors in a rat model using chronic corticosterone injections. Zhang et al. [31] demonstrated that the antidepressant effects of apigenin are associated with the promotion of autophagy via the mTOR/AMPK/ULK1 (mammalian target of rapamycin/AMP-activated protein kinase/Unc-51 Like Autophagy Activating Kinase 1) pathway in chronic-restraint-stressed mice. Can et al. [32] indicated that gallic acid exhibited an antidepressant-like effect in mice. Gallic acid seems to have a dual mechanism of action by increasing not only serotonin, but also catecholamine levels in synaptic clefts of the central nervous system. Wu et al. [32] reported the antidepressant potential of chlorogenic-acid-enriched extract from *Eucommia ulmoides* Oliver bark with neuron protection and promotion of serotonin release through the enhancement of synapsin I expression. Jin et al. [33] reported that rosmarinic acid ameliorated depression-like behaviors in a rat model of CUS (chronic unpredictable stress) and upregulated BDNF levels in the hippocampus and hippocampal-derived astrocytes. Anjaneyulu et al. [34] indicated that quercetin has the potential to be employed as a therapy for depression in streptozotocin-induced diabetic mice. De la Peña et al. [35] reported that luteolin mediated antidepressant-like effects in mice, possibly through modulation of the GABAA receptor (γ-aminobutiric acid type A receptors). Bedel et al. [36] demonstrated the antidepressant-like activity of ellagic acid and its effect on hippocampal-brain-derived neurotrophic factor levels in mouse depression models. 

Increased swimming time denotes a survival tendency that usually lacks in depressive individuals. The same attitude is characteristic for all living beings, not just for animals. Therefore, our extract effects are better than the impact of the positive control (TRM) and even better than the behavior of the healthy animals. Therefore, we may postulate that an enriched flavonoid diet may lower depressive behavior and increase life expectancy. 

## 3. Materials and Methods

### 3.1. Plant Material and Extraction Procedure

Aerial parts (leaves and flowers) of CC were collected between March and July 2017 from their natural habitat at Taounate, a northern province of Morocco. The macroscopic identification was performed by Professor Khalid Derraz, a botanist at the Faculty of Sciences and Techniques of Fez, Morocco. A voucher specimen was taxonomically identified and deposited in the herbarium of the Faculty of Sciences and Techniques, Fes, Morocco (MA-FSTF33). 

The fresh herbs were thoroughly washed individually under running tap water to remove any traces of soil particles and other dirt and dried in shade. The dried plant materials were powdered using a mechanical grinder before use. The percentage yields, based on the dried starting material, for the aqueous extract of CC condenses was 28% (*w*/*w*). For preservation, the liquid extract was lyophilized to a dry consistency and kept at 4 °C for further investigation.

### 3.2. Chemical Assessment of the Extract

The dried extract was solubilized in a methanol-water mixture with a ratio of 3:1. A 4 mg/mL stock solution was obtained and then filtrated through a Millipore syringe filter (0.2 µm pore diameter) and used for UHPLC (ultra-high-performance liquid chromatography) analysis. The system included a Thermo UltiMate 3000 chromatograph with a quaternary pump, an autosampler, a Phenomenex Luna Omega Polar C18 column (100 A, 150 × 4.6 mm), and a UV-DAD (multidiode array detector).

Briefly, the sample was injected in an amount of 10 µL and eluted with a mixture of acetonitrile with 0.1% phosphoric acid (A) and phosphoric acid 1% (B). The flow was established at 0.8 mL/min and the mixture elution was 0–10% A in B for 4 min, 10–15% for 4 min, linear for the next 4 min, then increased to 85% for 15 min, and back to 10% A for the last 3 min. The integration of the peaks and the calculation parameters were established using Chromeleon 7.2 v.12 software. Simultaneous chromatograms were registered at 280 nm, 330 nm, and 520 nm. Various standards (epicatechin, caffeic acid, rosmarinic acid, chlorogenic acid, ellagic acid, luteolin, apigenin, quercetin-3-arabinoside, apigenin-7-O-glucoside, and luteolin-7-O-glucoside) were used for identification of the compounds from the CC sample. Aliquots (2–10 µL) of the standard stock solutions (concentration ranges 0.114–0.195 mg/mL) were injected and analyzed in the same manner. For the external standards, calibration curves were obtained with a correlation coefficient above 0.9989. The standard deviation was calculated at 0.009. The limit of detection (LOD) and the limit of quantification (LOQ) of epicatechin and chlorogenic acid were calculated at 280 ng/mL and 145 ng/mL, respectively. The identification of the compounds found in the investigated sample was based on the retention time and UV spectra comparison with the standards for a match index above 950 from a total of 1000. 

All standards and solvents were of HPLC quality and were bought from Sigma Aldrich (Germany). The analysis was repeated three times and the quantification of the major compounds included in Table 1 represent the average value obtained from the arithmetic mean calculation of all triplicates.

### 3.3. Antioxidant Activity Evaluation

The antioxidant potential of the investigated CC extract was assessed by three methods: 2,2-diphenyl-1-picrylhydrazyl (DPPH) assay, iron chelation activity, and lipoxygenase (LOX) inhibition. These methods were previously described by Iancu et al. [16] and Gradinariu et al. [17]. 

For the DPPH assay, the decrease in the color intensity was measured at 517 nm with a UV spectrophotometer for 5 min. Forty microliters of each concentration sample (ranging from 1.25 mg/mL to 20 mg/mL) were added to the reaction mixture and the color changes were evaluated using methanol as a blank. 

On the other hand, the iron chelation activity was assessed at 562 nm using 80 µL ferrozine 5 mM solution in 0.1 M acetate buffer. The chelation intensity was established after 10 min of interaction between the investigated samples and the reaction mixture, kept in the absence of light. The absorbance measurement was evaluated against a blank and a control solution that contained 0.2 mL of ultrapure water, 0.74 mL of 0.1 M acetate buffer, and 0.02 mL of 2 mM ferrous sulfate solution in 0.2 M hydrochloric acid. To this mixture, 0.04 mL of 5 mM ferrozine solution was added after stirring for 10–15 s.

The lipoxygenase-inhibitory potential assay utilized a 0.05 mL solution of lipoxygenase in borate buffer mixed with 0.05 mL of CC extract in DMSO. After 10 min at room temperature, 2 mL of 0.16 mM linoleic acid solution in borate buffer was added. The absorbance of the solution was recorded at 234 nm, in the range of 0–120 s. A decrease in the absorbance intensity indicated the presence of antioxidant compounds in the sample. 

For all the assays, quercetin solution in similar concentrations was used as a standard. For easier interpretation, IC_50_ (inhibitory concentration of 50% of the DPPH/iron radicals or LOX present in the reaction mixture) was calculated by linear regression using the immediate values above and below 50% activity.

### 3.4. Animals

30 male Wistar rats (3 months old), weighing 250 ± 50 g, were held in a regulated environment (22 °C, 12-h light period beginning at 08:00 h) with free access to water and food. The rats were divided into six groups of five rats each, at random. For 14 days, the control group was given normal saline (1 mL/kg). The scopolamine (Sco) group was given Sco 30 min before the behavioral experiments, intraperitoneally (ip), at a dosage of 0.7 mg/kg body weight (bw). For 14 days, extract-treated groups were given Sco and then CC extract at 100 and 200 mg/kg ip. There were two additional groups: the diazepam group (DIAZ) [37] and the tramadol group (TRM) [38], used as positive controls for the elevated plus-maze and forced swimming tests. The doses of Sco and CC extract were chosen based on previously published studies [18,39]. 

All procedures were in accordance with Directive 2010/63/EU of the European Parliament and of the Council of 22 September 2010 on the protection of animals used for scientific purposes, and rats were handled according to animal bioethics guidelines from the Romanian Act on Animal Experimentation and Animal Health and Welfare.

The Faculty of Biology Animal Ethics Committee approved this report (protocol number No. 15309/22.07.2019), and attempts were made to mitigate animal suffering and reduce the number of animals used.

### 3.5. Elevated Plus-Maze (EPM)

The elevated plus-maze (designed by Coulbourne Instruments, Allentown, PA, USA) was used to assess anxiety levels. This black Plexiglas apparatus had two open arms opposite each other, two closed arms (49 × 10 cm) opposite each other, and a central sheath raised 50 cm above the surface. Each animal was gently positioned in the center of the device, facing the open arm, and allowed to explore for 5 min in a silent chamber. Rat behavior was filmed using a Logitech HD Webcam C922 Pro Stream camera and the videos were analyzed using ANY-maze software (designed by Stoelting Co., Wood Dale, IL, USA). The following variables were recorded: (1) the time spent in the open arms and the enclosed arms, and (2) the number of entries to any of the four arms. The entry of all four feet of the animal into one arm was described as an arm entry. The test lasted 5 min, and after the rat was removed and the apparatus was thoroughly washed with cotton and a 10% ethanol solution [40]. The reference drug in this study was diazepam, an anxiolytic agent.

### 3.6. The Forced Swimming Test (FST)

The forced swimming test (FST) is the most used animal model for depression. The model’s theoretical rationale is that uncontrolled stress causes behaviors that stimulate anhedonia, a common human depression syndrome. Rats were placed in a transparent cylindrical glass tank (height = 59 cm, internal diameter = 30 cm) containing water to a level of 25 cm (26 ± 1 °C). Water was changed for each rat. Rats were given a 15-min pretest swim session, followed by a 6-min test swim session the next day. Both swimming sessions took place from 12.00 to 18.00 h. The rats were taken out of the water, dried with towels, and put in a warm enclosure for 20 min before being returned to their home cages. The behavior of the rat was filmed using a Logitech HD Webcam C922 Pro Stream camera during a single exposure to forced swimming (6 min), and the videos were analyzed using ANY-maze software (from Stoelting Co., Wood Dale, IL, USA). Two forms of behavior were tested: (1) immobility (when the animal was immobile and floated in a straight position with only minor movements to hold its head above the water’s surface) and swimming (time spent with active swimming movements) [18]. Tramadol, an antidepressant and analgesic agent, was used as the reference drug in this study. 

### 3.7. Data Analysis

GraphPad Prism version 7.00 was used to analyze the data (GraphPad Software, La Jolla, CA, USA), by a one-way ANOVA with Tukey’s post hoc examination. The data were expressed as mean ± standard error of the mean (SEM) and a significance level of *p* < 0.05 was used. 

## 4. Conclusions

In summary, the present study suggested that the aerial parts of *C. canadensis* are rich in catechins and flavonoids, which sustain the antioxidant potential of the extracts used in therapy. Moreover, the CC extract acted as an anxiolytic and antidepressant agent in the Sco rat model by regulating the cholinergic system. Such results justify the therapeutic use of extracts obtained from CC. Furthermore, water is a nontoxic, easy-to-use, and reliable solvent for plant compounds. Our study refers only to the aqueous extracts and is limited to the compounds that were identified in them. However, further studies are needed to compare the extractability of various classes of substances synthesized by *Conyza* and pharmacokinetic evaluation is necessary to establish which compound is the main actor in neuroprotection.

## Figures and Tables

**Table 1:** Quantification of the major active compounds identified in *Conyza canadensis* aqueous extract.

No	Flavonoids (µg/mg Dry Extract)		Polyphenolic Acids (µg/mg Dry Extract)	
1	epicatechin	59.343 * ± 0.011	gallic acid	13.785 ± 0.011
2	catechin	40.818 ± 0.021	chlorogenic acid	3.243 ± 0.003
3	quercetin-3-arabinoside	4.650 ± 0.012	rosmarinic acid	24.557 ± 0.012
4	apigenin-7-O-glucoside	6.345 ± 0.012	ellagic acid	3.435 ± 0.012
5	luteolin-7-O-glucoside	3.440 ± 0.005		
6	luteolin	26.308 ± 0.012		
7	apigenin	6.170 ± 0.021		
8	quercetin	6.468 ± 0.012		
	Total identified	153.542 ± 0.022		45.020 ± 0.023

* Values included in the table represent the mean of triplicate quantification; limit of detection (LOD): 280 ng/mL; limit of quantification (LOQ): 145 ng/mL.

## Data Availability

The data presented in this study are available on request from the corresponding author.
